# Leiomyosarcoma with partial rhabdomyoblastic differentiation: First case report of primary cardiac origin

**DOI:** 10.1186/1471-2407-11-76

**Published:** 2011-02-17

**Authors:** Yoichiro Okubo, Kazutoshi Shibuya, Atsushi Namiki, Kazuhisa Takamura, Noriaki Kameda, Tetsuo Nemoto, Aki Mitsuda, Megumi Wakayama, Minoru Shinozaki, Nobuyuki Hiruta, Kanako Kitahara, Takao Ishiwatari, Junichi Yamazaki

**Affiliations:** 1 Department of Surgical Pathology, Toho University School of Medicine, 6-11-1 Omori-Nishi, Ota-Ku, Tokyo, 143-8541, Japan; 2 Division of Cardiovascular Medicine, Department of Internal Medicine, Toho University Omori Medical Center, Toho University School of Medicine, 6-11-1 Omori-Nishi, Ota-Ku, Tokyo, 143-8541, Japan

## Abstract

**Background:**

Leiomyosarcoma occurring as a primary cardiac tumor has been known as an extremely rare condition. Previous studies of leiomyosarcoma with rhabdomyoblastic differentiation have conducted to those arisen from another site, and they indicated a poorer prognosis of this tumor.

**Case presentation:**

A 69-year-old woman was referred to our hospital for an operation concerning umbilical hernia. Subsequent imaging examinations before an operation indicated the presence of primary cardiac malignant tumor due to its atypical shape. And then, it was surgically removed. Histopathologically, tumor cells consisted of two different types: spindle and polyhedral cells. Immunohistochemically, it is interesting to note that 2.1% of spindle cells and 23.1% of polyhedral cells showed positive reactivity for myogenin. Furthermore, we performed double-immunostaining for alpha-smooth muscle actin (SMA) and myogenin. The rates of alpha-SMA and myogenin double negative, alpha-SMA single positive, myogenin single positive, and alpha-SMA and myogenin double positive in spindle cells were estimated as 69.1%, 28.8%, 1.1% and 1.0%, respectively. In contrast, the rates in polyhedral cells were estimated as 76.9%, 0.0%, 23.1%, and 0.0%, respectively.

**Conclusion:**

Our immunohistochemical evaluation suggested that rhabdomyoblastic differentiation in leiomyosarcoma might be generated not only by de novo generation from mesenchymal cells. To the best of our knowledge, this is the first case of primary cardiac leiomyosarcoma with partial rhabdomyoblastic differentiation.

## Background

Primary cardiac tumors represent a rare neoplastic condition with an incidence that ranges from 0.0017 to 0.019% [1], of which 25% are malignant. Among such tumors, angiosarcoma is the commonest malignant tumor followed by rhabdomyosarcoma, malignant mesothelioma, and fibrosarcoma, each with an incidence that is greater than 10% [2]. However, the incidence of cardiac leiomyosarcoma is less than 1% [2]. Previous studies of leiomyosarcoma with rhabdomyoblastic differentiation have conducted to those arisen from another site [3-11], and they announced a poorer prognosis of this tumor. Especially, Oshiro et al. have reported that leiomyosarcoma with rhabdomyoblastic differentiation shows poorer prognosis than typical leiomyosarcoma [6]. In the present paper, we describe an extremely rare primary cardiac malignant tumor. To the best of our knowledge, this is the first case of primary cardiac leiomyosarcoma with partial rhabdomyoblastic differentiation.

### Case presentation

**Figure 1:**
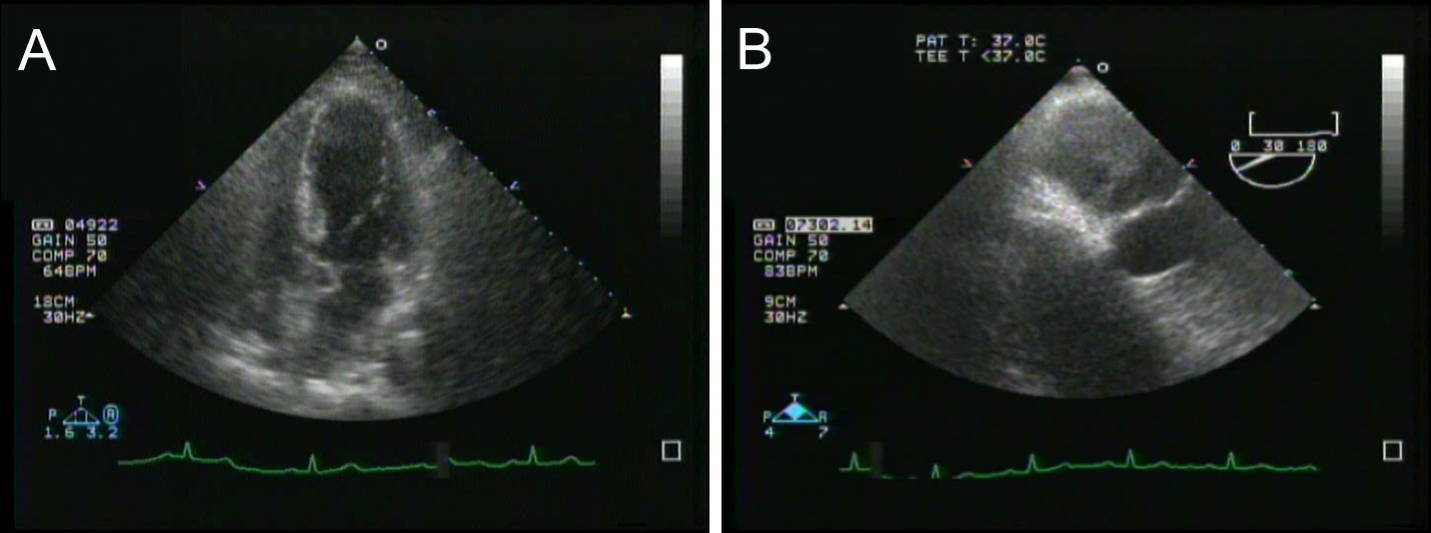
**Photograph showing cardiac ultrasonography**. (A) Transthoracic cardiac ultrasonography performed in our hospital showed showing a club -shaped tumor of 34 mm in diameter inside the left atrial cavity in a four-cavities tomogram. (B) Transesophageal cardiac ultrasonography showed showing a broad-based, gigantic, and multilocular tumor occupying almost the entire left atrium.

A 69-year-old woman was referred to our hospital for an operation concerning umbilical hernia who had been diagnosed with hypertension and polycystic kidney disease one year prior to her surgery. Subsequent transthoracic cardiac ultrasonography in our hospital showed a club-shaped tumor of 34 mm in diameter inside the left atrial cavity in a four-cavities tomogram. Transesophageal cardiac ultrasonography showed a broad-based, gigantic, and multilocular tumor occupying almost the entire left atrium (Figure 1). Chest computed tomography (CT) showed no abnormality in the lungs or hilar lymph nodes. Abdominal CT showed multilocular cysts in bilateral kidneys. Cardiac magnetic resonance imaging showed a broad-based protuberant tumor which had a T1 iso-signal intensity and high T2 signal intensity in the posterior wall of the left atrium. Positron emission tomography analysis showed abnormal 18F- fluorodeoxy glucose uptake which was detected only in the heart, with the exception of the umbilical hernia lesion. These results indicated the presence of primary cardiac malignant tumor due to its atypical shape. Finally, surgical removal with the patient's permission was performed. Almost all of the tumor could be removed and subsequent chemotherapy was considered. However, the patient's renal dysfunction ruined adjuvant chemotherapy and she died of her disease nine months after the surgical removal due to multiple lung metastases.

### Pathologic findings

**Figure 2:**
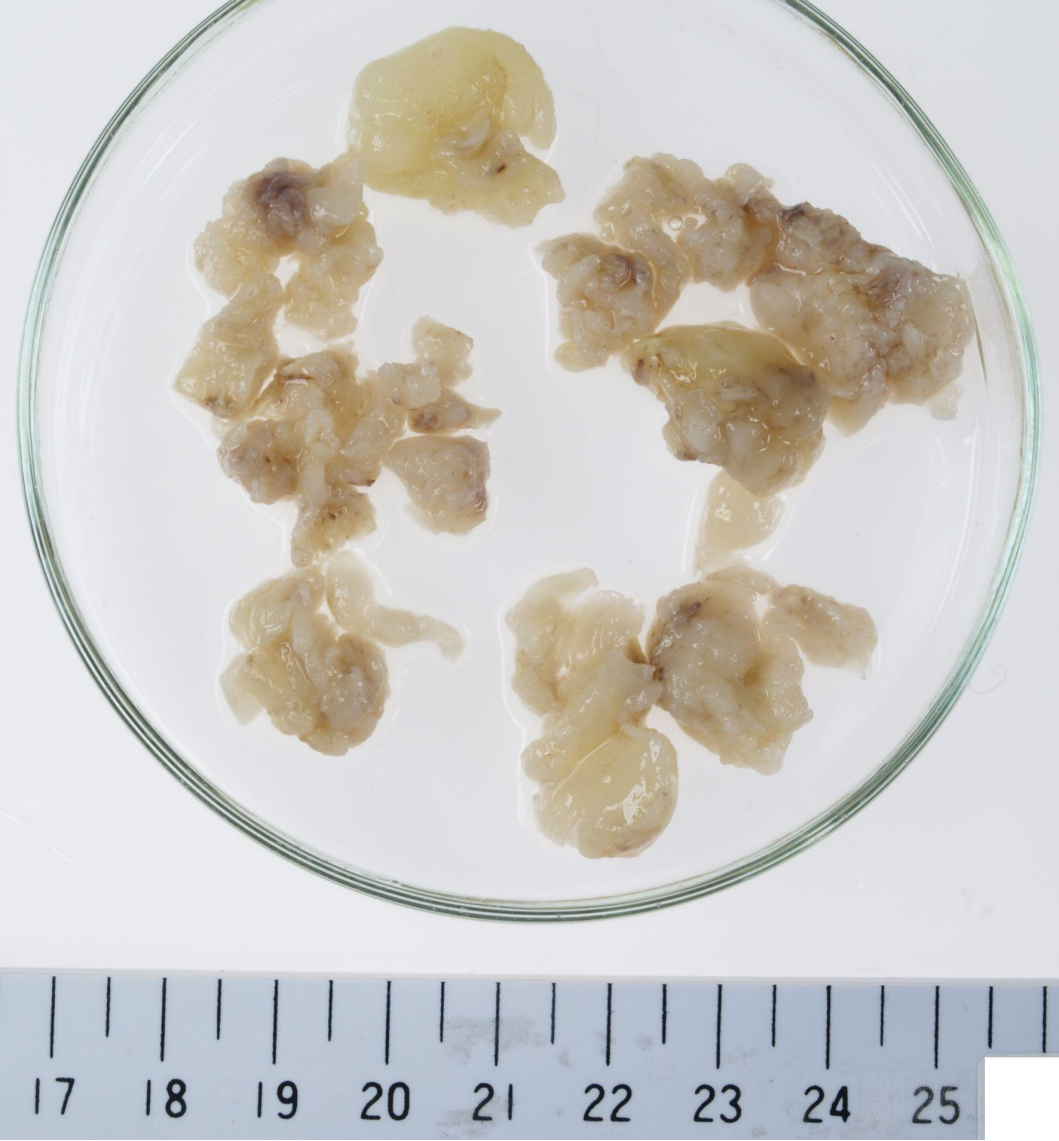
**Photograph of the surgical specimen**. The submitted specimen comprised several cakes of the tumor (measuring up to 41 × 28 × 14 mm in size) with a gray-white color on the surface.

Macroscopically, the submitted specimen comprised several cakes of the tumor with a gray-white color on the surface (Figure 2). It was fixed with 10% buffered formalin, embedded in paraffin wax after dehydration, and cut into four μm-thick sections. These were then prepared and stained with hematoxylin and eosin (HE) double stain for light microscopic observation.

**Figure 3:**
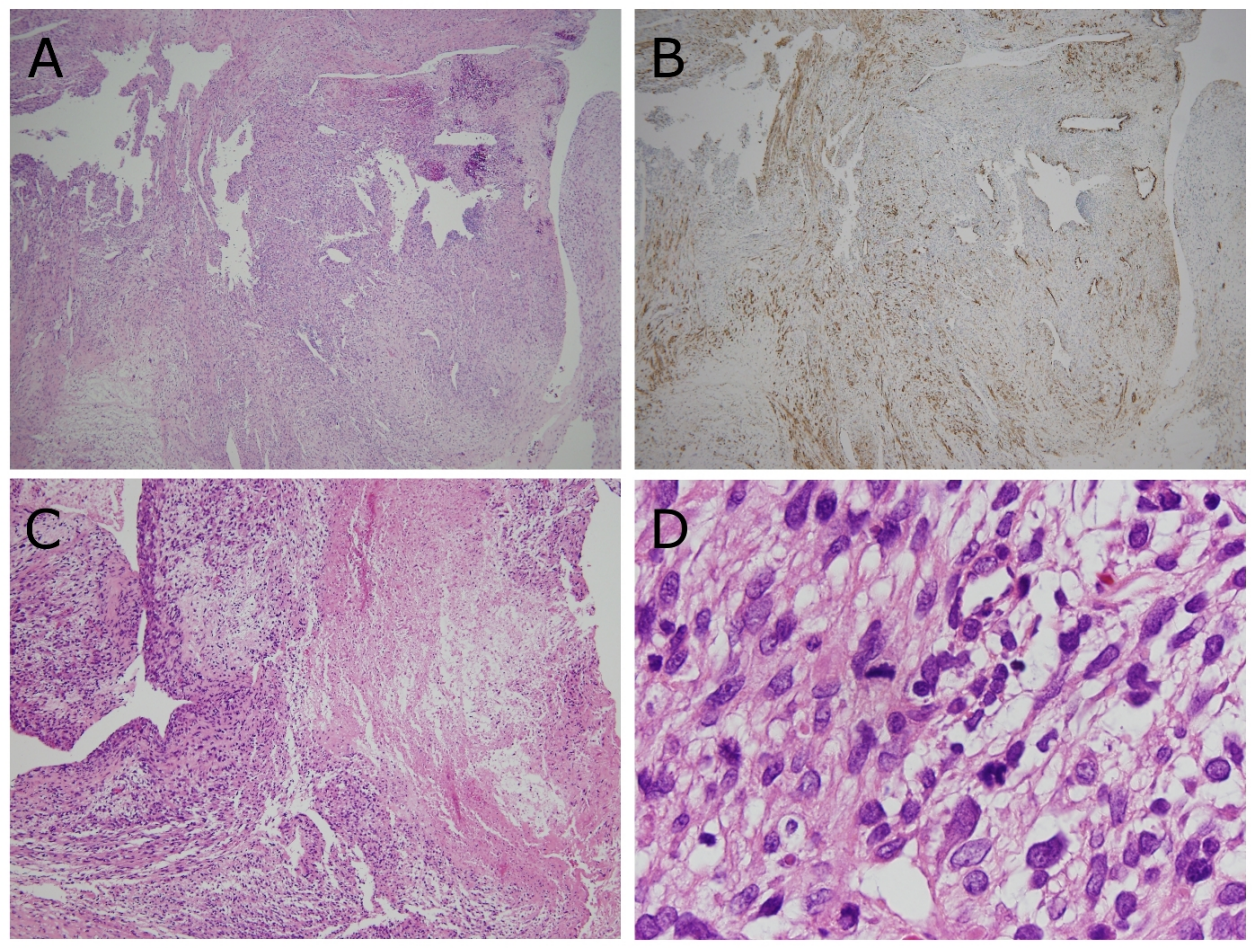
**Photomicrographs showing spindle cells with areas of hypercellularity or necrosis**. (A) Low-power view of the area with spindle cells proliferation (Hematoxylin and Eosin (HE) double stain, × 40). (B) Spindle cells with positive reactivity for α-smooth muscle actin (SMA) comprise approximately 30% area of the tumor (Immunostain, anti-α-SMA × 40). (C) There is focus of necrosis in the limited the tumor. Following to the French National Federation of Cancer grading system, presence of tumor necrosis less than half in area corresponds to score-1 (HE double stain, × 100). (D) Spindle cells showed mitosis. On average, twelve mitoses per ten high-power fields were confirmed. Following to the French National Federation of Cancer grading system, mitotic activity of the present case corresponds to score-2 (HE double stain, × 1000).

**Figure 4:**
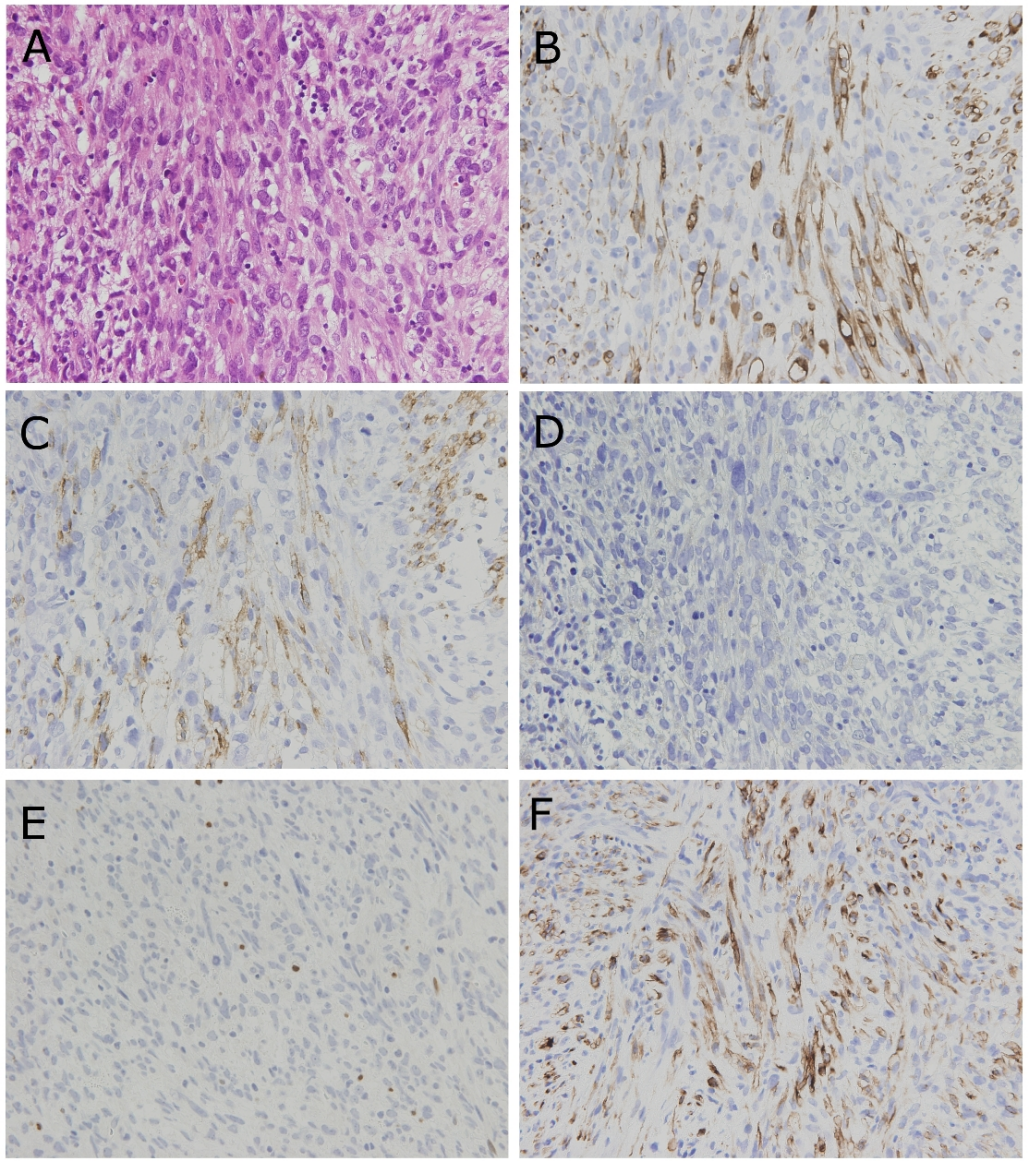
**Photomicrographs showing spindle cells and their phenotypical expression**. (A) Spindle cells had an elongated, blunt-ended and hyperchromatic nucleus plus spindle, were fibrillated, and possessed an eosinophilic cytoplasm. Occasional cells have perinuclear vacuoles (Hematoxylin and Eosin double stain, × 400). (B, C, D, E, and F) Photomicrographs of immunostain with desmin (B), α-smooth muscle actin (C), myoglobin (D), myogenin (E), and cytokeratin CAM 5.2 (F), respectively (× 400).

**Figure 5:**
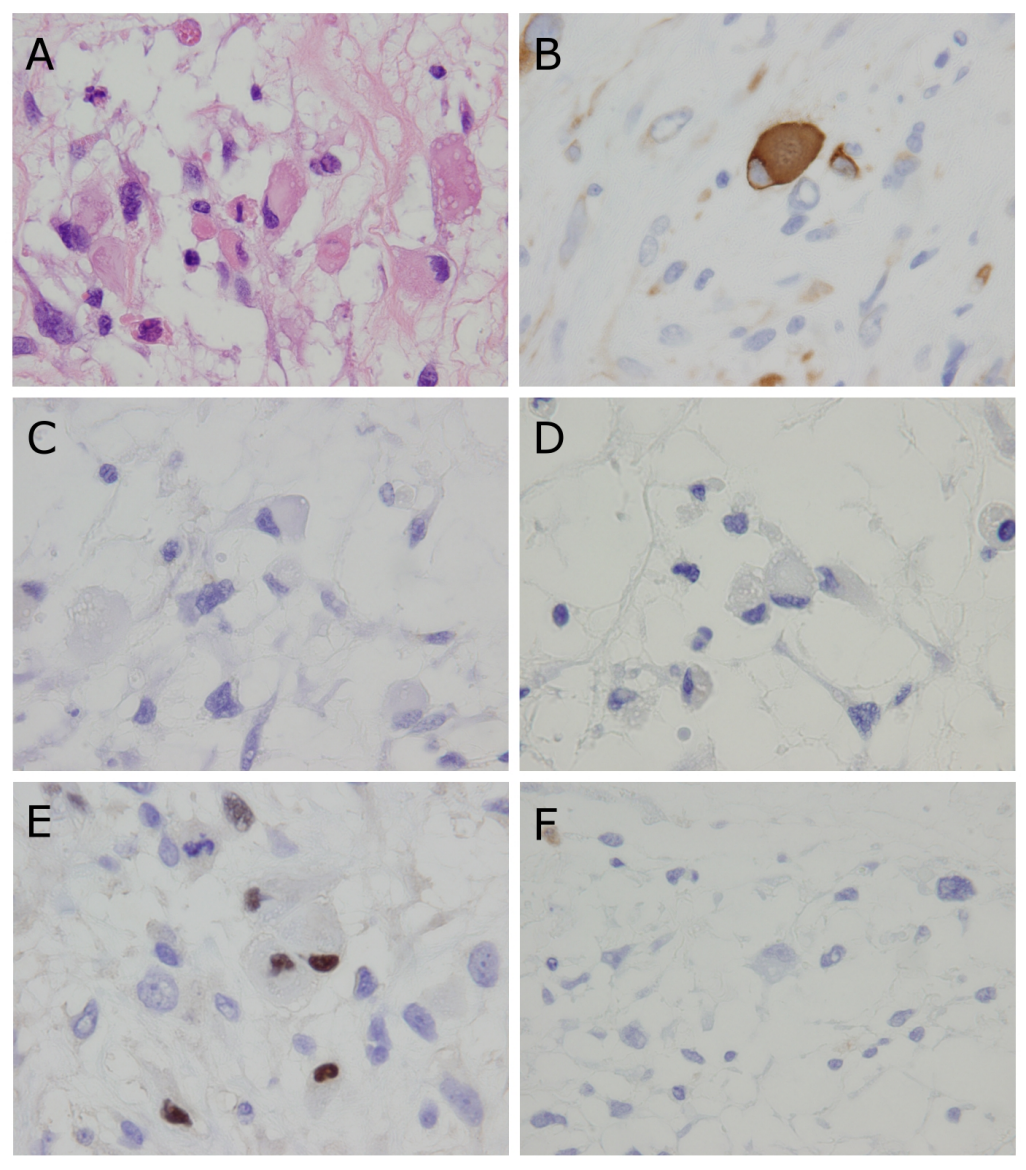
**Photomicrographs showing polyhedral cells and their phenotypical expression**. (A) Polyhedral cells had a hyperchromatic and eccentric nucleus with a polyhedral, large, and eosinophilic cytoplasm (Hematoxylin and Eosin double stain, × 1000). (B, C, D, E, and F) Photomicrographs of immunostain with desmin (B), α-smooth muscle actin (C), myoglobin (D), myogenin (E), and cytokeratin CAM 5.2 (F), respectively (× 1,000).

Histopathologically, tumor cells that had proliferated in the myxoedematous matrix (Figure 3A) consisted of two different types: a large portion comprised spindle cells, and polyhedral cells were also identified as a minor component. Spindle cells had an elongated, blunt-ended and hyperchromatic nucleus plus spindle, were fibrillated and possessed an eosinophilic cytoplasm (Figure 3D and 4A). In contrast, polyhedral cells had a hyperchromatic and eccentric nucleus with a polyhedral, large, and eosinophilic cytoplasm (Figure 5A). Spindle cells showed twelve mitoses per ten high-power fields. Accordingly, the histological grade of the tumor corresponded to grade-2 (tumor differentiation: score-2; mitotic counts: score-2; tumor necrosis: score-1) following to the French National Federation of Cancer Centers (FNCLCC) grading system (Figure 3C and 3D) [12].

Although a few polyhedral cells were confirmed, morphological findings on HE double stain indicated a myxoid type of leiomyosarcoma.

### Immunohistochemical findings

**Figure 6:**
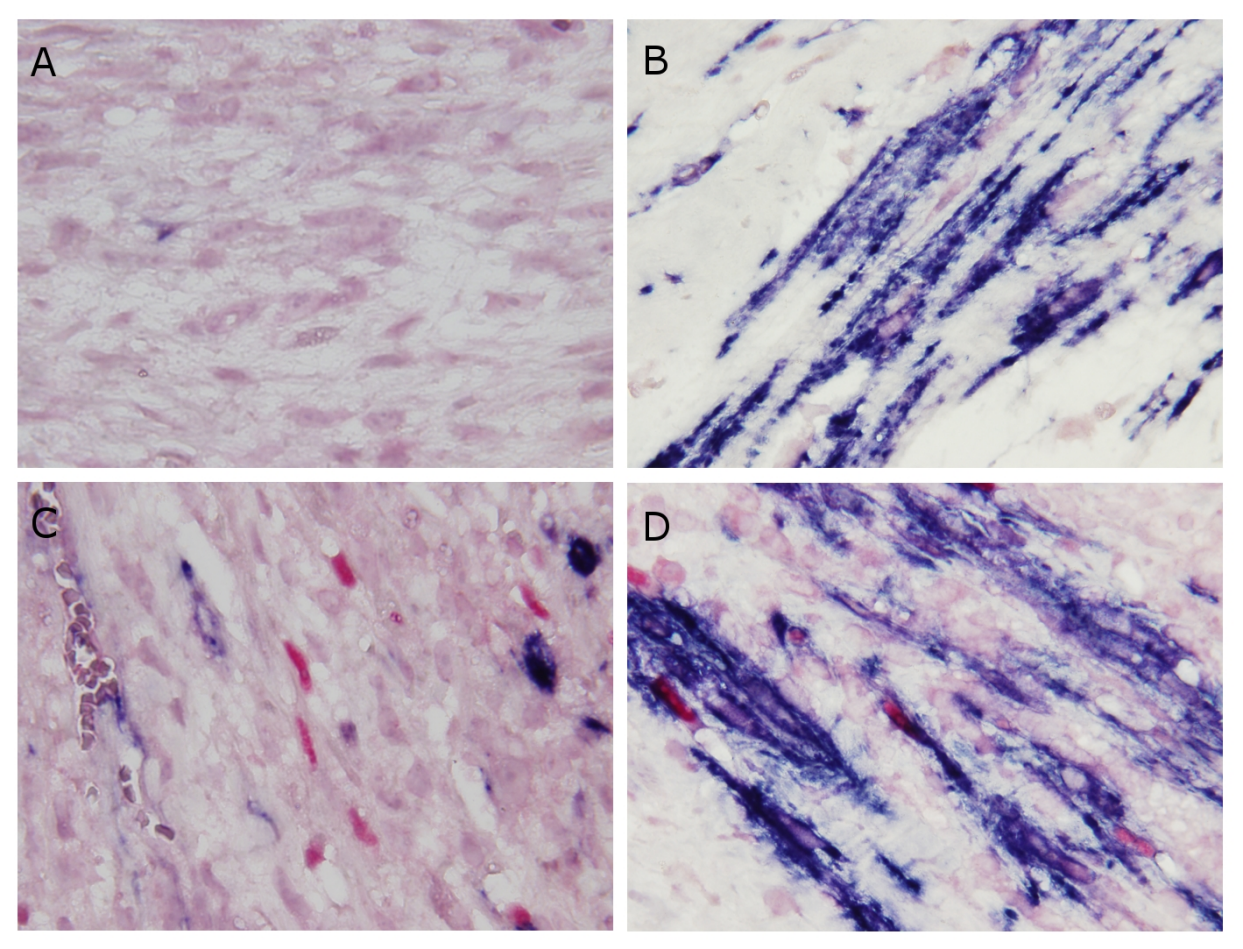
**Photomicrograph showing the results of double-immunostaining of spindle cells**. Positive reactivity for α-smooth muscle actin (SMA) is recognized as a blue immunohistochemical signal in the cytoplasm, and for myogenin is recognized as a red immunohistochemical signal in the nucleus. (A) Negative reactivity for both α-SMA and myogenin (× 1000). (B) Positive reactivity for α-SMA alone (× 1000). (C) Positive reactivity for myogenin alone (× 1000). (D) Positive reactivity for both α-SMA and myogenin (× 1000).

Several kinds of monoclonal antibody were used to evaluate tumor cells immunohistochemically and included anti-Vimentin, CD31, CD34, cytokeratin (CK AE1/AE3, 34 β-E 12, 5/6, and CAM 5.2), desmin, α-smooth muscle actin (α-SMA), myoglobin, myogenin, and Ki-67 (MIB-1) antibodies. All tumor cells showed strong positive reactivity for vimentin and negative reactivity for CD31, CD34, and myoglobin (Figure 4D and 5D). Spindle cells showed focal positive reactivity for desmin, α-SMA, and cytokeratin CAM 5.2 (Figure 4B, C, and 4F). In contrast, polyhedral cells showed positive reactivity for desmin, but negative reactivity for α-SMA and cytokeratin CAM 5.2 (Figure 5B, C, and 5F). Ki-67 (MIB-1) labeling index in the spindle and polyhedral cells were estimated as 27.1% and 33.3%, respectively. It is interesting to note that 2.1% of spindle cells and 23.1% of polyhedral cells showed positive reactivity for myogenin (Figure 4E and 5E). Furthermore, to determine whether tumor cells are present as double positive for both α-SMA and myogenin, and to ascertain the morphological characteristics of these tumor cells, we performed double-immunostaining for α-SMA and myogenin. The rates of α-SMA and myogenin double negative, α-SMA single positive, myogenin single positive, and α-SMA and myogenin double positive in spindle cells were estimated as 69.1%, 28.8%, 1.1%, and 1.0%, respectively (Figure 6A, B, C, and 6D). In contrast, the rates in polyhedral cells were estimated as 76.9%, 0.0%, 23.1%, and 0.0%, respectively. These results are summarized in Table 1. According to the morphological and immunohistochemical findings, we diagnosed this primary cardiac tumor as a leiomyosarcoma with partial rhabdomyoblastic differentiation.

**Table 1:** Summary of phenotypical expression by immunohistochemical examination

	Spindle cell	Polyhedral cell
α-SMA and myogenin double negative α-SMA (-) and myogenin (-)	69.1%	76.9%
α-SMA single positive α-SMA (+) and myogenin (-)	28.8%	0.0%
myogenin single positive α-SMA (-) and myogenin (+)	1.1%	23.1%
α-SMA and myogenin double positive α-SMA (+) and myogenin (+)	1.0%	0.0%

SMA: smooth muscle actin

The rates of α-SMA and myogenin double negative, α-SMA single positive, myogenin single positive, and α-SMA and myogenin double positive in spindle cells were estimated as 69.1%, 28.8%, 1.1% and 1.0%, respectively.

## Discussion

Leiomyosarcoma occurring as a primary cardiac tumor has been known as an extremely rare condition of which the rate represents less than 1% of all primary cardiac malignant tumors [2]. Furthermore, to the best of our knowledge there has been no report of a case of primary cardiac leiomyosarcoma with partial rhabdomyoblastic differentiation. In general, leiomyosarcoma is currently subdivided histologically into four types: classical, epithelioid, pleomorphic, and myxoid [13]. The morphological findings of the present case indicated a myxoid type of leiomyosarcoma, but immunohistochemistry revealed that a few tumor cells showed positive reactivity for myogenin. This has been known as a myogenic transcriptional regulatory protein which is expressed in the early phase of skeletal muscle differentiation (rhabdomyogenic differentiation), and it induces differentiation of myoblasts into the multinucleated myotube [14]. This myogenic regulatory protein has been largely accepted as a sensitive and specific immunohistochemical marker for rhabdomyosarcoma or other tumors with rhabdomyoblastic differentiation [14].

Meanwhile, it is interesting to note that the spindle cell showed positive reactivity for CK CAM 5.2. Although, it has been well known that leiomyosarcoma usually showed negative reactivity for epithelial markers [15], some investigators described that a part of leiomyosarcoma shows positive reactivity for CK [15-17]. Therefore, CK CAM 5.2 expression in the present case may support a diagnosis of leiomyosarcoma. However, to make diagnosis of leiomyosarcoma with rhabdomyoblastic differentiation, we should refer three important tumors and deny them, respectively, which comprise undifferentiated pleomorphic sarcoma (UPS), rhabdomyosarcoma, and rhabdomyoma. Cardiac UPS usually occurring at the left atrium, histopathologically comprises a mixture of spindle cells in a storiform pattern with polyhedral cells [5]. Furthermore, high-grade undifferentiated sarcomas can exhibit focal α-SMA expression [15]. These findings are similar to the present case. However, the spindle cell, a major component of the present tumor, had an elongated, blunt-ended, and hyperchromatic nucleus plus spindle, fibrillated, and eosinophilic cytoplasm. In addition, the cell showed positive reactivity both for α-SMA and desmin, focally, by immunohistochemical examination. These findings allowed disclosing the smooth muscle differentiation. Furthermore, some of the spindle cell also showed positive reactivity for CK CAM 5.2, of which positive ratio has been reported ranging from 22.2% (2/9) to 35.0% (14/40) in leiomyosarcoma [16,17]. Although it still remains a difficulty for decision, we made the diagnosis of leiomyosarcoma rather than UPS. On the other hand, since rhabdomyosarcoma has been know as the second most common primary cardiac malignant tumor [2], that should also be considered as a disease for differential diagnosis. Especially, embryonal rhabdomyosarcoma usually shows similar morphologic findings of the present case, such as varying degrees of cellularity containing hypercellular and loosely textured myxoid areas, hyperchromatic and round or spindle-shaped nucleus, and eosinophilic cytoplasm [18]. However, embryonal rhabdomyosarcoma is uncommon in patients older than 40 years of age [18] and neither cross-striation nor myoglobin expression was proven in the present case. Furthermore, a large body of spindle cells showed negative reactivity for myogenin (only 2.1% of them showed positive reactivity) that has been largely accepted as a sensitive and specific immunohistochemical marker for rhabdomyosarcoma or other tumors with rhabdomyoblastic differentiation [14]. According to our immunohistochemical examinations, we were able to deny typical rhabdomyosarcoma. As for rhabdomyoma, the most common subtype of cardiac origin has been known as cardiac rhabdomyoma, but it occurs almost exclusively in the hearts of infants and young children and composes predominantly large polygonal vacuolated spider cells [19]. Therefore, the adult type of rhabdomyoma should be considered as differential diagnosis which is usually composed of tightly polygonal cells which had peripherally placed nucleus plus acidophilic, finely granular, and vacuolated cytoplasm. However, mitotic figures are nearly absent, cross-striations can be discerned, and show positive reactivity for rhabdomyogenic markers immunohistochemically in these two subtypes of rhabdomyoma [19]. These results were different from the findings extracted from the present case.

On the other hand, only one case of sarcoma arisen from myocardium with rhabdomyoblastic differentiation has been reported by Kabir et al. [20] who described a malignant peripheral nerve sheath tumor indicated an area of rhabdomyoblastic differentiation in part. In their report, a little information of immunohistochemical examinations was described which simply comprised positive reactivity for s-100 protein and focal for desmin. These results were different from these of the present case, but comparative discussion could not be completed in detail. Therefore, we preferred to diagnose this primary cardiac malignant tumor as a leiomyosarcoma with partial rhabdomyoblastic differentiation. Previous studies of leiomyosarcoma with rhabdomyoblastic differentiation have conducted to those arisen from another site, and Oshiro et al. have reported that leiomyosarcoma with rhabdomyoblastic differentiation shows poorer prognosis than typical leiomyosarcoma [6]. In fact, the present case showed rapid growth of the tumor and the patient died due to extensive metastases in the lungs despite early diagnosis and surgical removal. Table 2 presented herein summarizes major clinical data of nineteen cases of leiomyosarcoma in soft tissue with rhabdomyoblastic differentiation, and includes the present case representing the first report of leiomyosarcoma arising from the myocardium [table 2]. The patient age ranged from 33 to 85 (mean: 62.7). The male-to-female ratio was 10:10. The tumor sizes ranged from 20 to 250 mm (mean: 116.4).

**Table 2:** Literature summary of cases of leiomyosarcoma with rhabdomyoblastic differentiation

Reference	Year	Age (years)	Sex	Site	Size (mm)	Operation and adjuvant therapy	Follow up
Falconieri et al [3].	1996	83	Female	Breast	60	Radical mastectomy	10 mo NED
Roncaroli et al [4].	1996	59	Female	Retroperitoneum	170	Excision	8 mo NED
Leoong et al [5].	1996	56	Male	Stomach	60	Partial gastrectomy	NR
Oshiro et al [6].	2000	55	Female	Abdominal cavity	180	Marginal excision	85 mo NED
Oshiro et al [6].	2000	62	Female	Omentum	130	Marginal excision	NR
Oshiro et al [6].	2000	53	Female	Thigh, subcutis	80	Wide excision	19 mo DOD
Oshiro et al [6].	2000	76	Male	Buttock, subcutis	60	Wide excision	27 mo NED
Oshiro et al [6].	2000	33	Male	Thigh muscle	60	Wide excision	45 mo DOD
Oshiro et al [6].	2000	54	Male	Thigh muscle	220	Wide excision and chemotherapy	Lung metastasis, 9 mo DOD
Oshiro et al [6].	2000	84	Male	Buttock	30	Wide excision, radiation, and chemotherapy	93mo NED
Oda et al [7].	2001	50	Female	Back	NR	Excision	6 mo DOD
Oda et al [7].	2001	60	Male	Retroperitoneum	140	Excision	NR
Oda et al [7].	2001	85	Male	Buttock	20	Excision	5 mo local recurrence (additional wide excision), 65 mo NED
Oda et al [7].	2001	33	Male	Thigh	60	Wide excision and chemotherapy	Lung metastasis, 45 mo DOD
Oda et al [7].	2001	76	Male	Buttock	60	Excision	NR
Levine et al [8].	2002	72	Female	Uterus	210	Hysterectomy with salpingo-oophorectomy	6 mo NED
Shintaku et al [9].	2004	70	Female	Uterus	250	Hysterectomy with salpingo-oophorectomy and chemotherapy	Liver metastasis, outcome was NR
Nikaido et al [10].	2004	67	Male	Inferior vena cava	140	Radical excision	Lung metastasis, 13 mo DOD
Yorulmaz et al [11].	2007	56	Female	Uterus	240	Surgical removal, radiation, and chemotherapy	8 mo DOD
Present case	2009	69	Female	Heart	41	Surgical removal	Lung metastasis, 9 mo DOD

NR: not reported, DOD: died of disease, NED: no evidence of disease, mo: months

There are twenty cases of leiomyosarcoma with rhabdoid differentiation including the present case.

We wish to take a more detailed discussion on the present case, especially in relation to tumor cell differentiation with phenotypical expression analysis. The spindle cell, a major component of the tumor, has the potential to differentiate into a smooth muscle cell which can be phenotypically identified with positive reactivity for α-SMA. However, we found 2.1% of spindle cells showed positive reactivity for myogenin, and half of myogenin-positive spindle cells showed positive reactivity for α-SMA at the same time. In contrast, none of polyhedral cells showed positive reactivity for α-SMA, but they exhibited a significantly higher myogenin-positive rate than spindle cells. These findings support the following hypothesis. First, the "rhabdomyoblastic differentiation" observed in the present case may represent the early stage of rhabdomyogenic differentiation because tumor cells showed positive reactivity for myogenin which has been known as a maker of cells in the early phase of rhabdomyogenic differentiation, and exhibited neither cross-striation nor myoglobin expression. Second, polyhedral cells may be in a more advanced stage of rhabdomyogenic differentiation than spindle cells because polyhedral cells were morphologically similar to rhabdomyoblasts and showed a significantly higher myogenin-positive rate than spindle cells.

Finally, the possibility of transdifferentiation or synchronous smooth and skeletal muscle differentiation in leiomyosarcoma was suggested in the present case because synchronous expression of α-SMA and myogenin was confirmed in 1.0% of spindle cells. Overall, our immunohistochemical evaluation indicated that rhabdomyoblastic differentiation in leiomyosarcoma might be generated not only by de novo generation from mesenchymal cells.

## Conclusion

We describe an extremely rare case of primary cardiac leiomyosarcoma with partial rhabdomyoblastic differentiation. The tumor indicated aggressive growth and the patient died despite early diagnosis and surgical removal. Furthermore, our immunohistochemical evaluation suggested that rhabdomyoblastic differentiation in leiomyosarcoma might be generated not only by de novo generation from mesenchymal cells. To the best of our knowledge, this is the first report of primary cardiac leiomyosarcoma with partial rhabdomyoblastic differentiation.

## Consent

We could not get the proof of the patient's written and signed consent for the publication because we could not announce disease of our patient to herself due to her family's request. Furthermore, the patient has already died. However her family agreed our proposal using surgical specimen for our research and written informed consent was obtained from the patient's family (as a proxy) for publication of this case report and any accompanying images. A copy of the written consent is available for review by the Editor-in-Chief of this journal.

## Competing interests

Dr. Shibuya reports receiving research grants from Pfizer Japan Inc., Janssen Pharmaceutical K.K., and Dainippon Sumitomo Pharma Co. All authors declare that they have no competing interests.

## Authors' contributions

YO conceptualized the case report, integrated the data, and wrote the manuscript as a major contributor; KS carried out the histopathological evaluation and revised the manuscript; AN contributed to management of the patient and revised clinical description; KT contributed to management of the patient and gave final approval to the manuscript as a corresponding author; NK, TN, AM, and MW carried out the histopathologic evaluation and revised histopathological description; MS, NH, KK, and IT carried out the histopathologic evaluation, JY contributed to management of the patient as a chief doctor of Division of Cardiovascular Medicine. All authors have read and approved the final manuscript.

## Pre-publication history

The pre-publication history for this paper can be accessed here:

http://www.biomedcentral.com/1471-2407/11/76/prepub
